# Management and outcomes of catatonia: A prospective study in urban South Africa

**DOI:** 10.1177/20503121221105579

**Published:** 2022-06-20

**Authors:** Zukiswa Zingela, Louise Stroud, Johan Cronje, Max Fink, Stephan van Wyk

**Affiliations:** 1 Executive Dean’s Office, Nelson Mandela University, Gqeberha, South Africa; 2 Department of Psychology, Nelson Mandela University, Gqeberha, South Africa; 3 Stony Brook University, Stony Brook, NY, USA; 4 Department of Psychiatry and Human Behavioural Sciences, Walter Sisulu University, Mthatha, South Africa; 5 Nelson Mandela Academic Hospital, Mthatha, South Africa

**Keywords:** Catatonia, mental health, management, lorazepam, electroconvulsive therapy

## Abstract

**Objectives:**

Rapid intervention for catatonia with benzodiazepines and electroconvulsive therapy can prevent fatal complications. We describe the management and treatment response of 44 patients with catatonia in a psychiatric unit in urban South Africa. The objective was to screen admissions for catatonia and investigate management, treatment response, and treatment outcomes.

**Method:**

We used a prospective, descriptive, observational study design and collected data using a data collection sheet, the Bush Francis Catatonia Screening Instrument, the Bush Francis Catatonia Rating Scale, and the *Diagnostic Statistical Manual*-5 to assess catatonia in new admissions from September 2020 to August 2021.

**Results:**

Of the 241 participants screened on admission, 44 (18.3% of 241) screened positive for catatonia on the Bush Francis Catatonia Screening Instrument, while 197 (81.7% of 241) did not. Thirty-eight (86.4% of 44) received lorazepam, seven (15.9%) received clonazepam, and two (4.6%) received diazepam, implying that three (6.8%) of the 44 participants with catatonia received more than one benzodiazepine sequentially. Ten (22.7% of 44) patients received electroconvulsive therapy. Seven of those treated with electroconvulsive therapy (15.9% of 44 and 70% of 10) responded well and were discharged, whereas 22 (50% of 44 and 64.7% of 34) of those given lorazepam were discharged. Patients treated with electroconvulsive therapy had a higher initial Bush Francis Catatonia Rating Scale score. One patient (2.3%) relapsed within 4 weeks of discharge. Twenty (45.5%) of the 44 patients with catatonia had low average iron levels, 14 (31.8%) had low vitamin B12, and 24 (54.6%) had high creatinine kinase.

**Conclusion:**

Both lorazepam and electroconvulsive therapy were found to be effective treatments for catatonia with good response and outcomes. The length of hospital stay of patients with catatonia was similar to that of patients without catatonia. Treatment guidelines for catatonia need to include the role and timing of electroconvulsive therapy to augment current treatment protocols for the use of lorazepam.

## Introduction

The prevalence rate of catatonia indicates that it is not rare,^1–5^ with a prevalence range of 11.9% to 18.3% reported at the study site.^6,7^ The main signs and symptoms of catatonia are psychological or motor, with motor signs presenting as excessive slowing down or motor agitation to the point of excitement.^8–14^ Autonomic instability may also occur with changes in pulse rate, blood pressure, and body temperature.^10–12,14–21^

The causes of catatonia include severe mental disorders or medical illness such as meningitis or encephalitis; head trauma or space-occupying lesions, kidney, liver, and lung abnormalities, diabetes, thyroid disorders; cardiac failure,^1,12,14,19,20,22–26^ and substances in up to 25% of cases.^4,17,18,27^ Biological drivers of catatonia involve deficient gamma-aminobutyric acid (GABA)-modulated functions in the orbitofrontal cortex of the human brain.^4,28^ Benzodiazepines (BZDs) have a GABA receptor modulatory effect and are effective in treating catatonia.^4^ GABA-A binding is also reduced in the cortical areas of patients with catatonia, and this has been correlated with the motor and affective symptoms of catatonia, which revert to normal after exposure to lorazepam.^28^

Since BZDs are powerful anxiolytics and are an effective treatment for catatonia.^13,17,19^ This points to underlying anxiety as another key factor in catatonia.^13,27,29,30,31^ The effectiveness of BZDs in relieving catatonia is further supported by the fact that most projection neurons in the basal ganglia, which are crucial for motor function, are GABAergic.^13^ In addition, abrupt BZD withdrawal may precipitate catatonia.^22^ The overlap in the signs of catatonia and Parkinson’s also raises the possibility of similar dysfunction in the basal ganglia.^13^ Catatonia also occurs in anti-*N*-methyl-d-aspartate receptor encephalitis (A-NMDA-E), which is due to autoantibodies against NMDA receptors that are crucial for motor and emotional functions.^32,33^

Protracted catatonia is associated with poor treatment response, while lower scores on catatonia rating scales and an acute onset have been linked to better responses.^1,4,5,24^ Complications of catatonia include malnutrition, vitamin deficiencies, autonomic instability, aspiration pneumonia, urinary tract infection, dehydration, electrolyte imbalances, renal failure, and pulmonary emboli, all of which may be fatal if untreated.^1,4,34–38^ Response to treatment is good when interventions with effective treatments like BZDs and electroconvulsive therapy (ECT) are instituted early.^1,4,36–42^ Concurrent use of ECT and BZD is appropriate in severe cases, and a BZD antagonist, flumazenil, is useful for addressing the anti-seizure effects of lorazepam while administering ECT.^1–5^

Short-acting BZDs are particularly effective in the treatment of catatonia^1,4,30–33,43–47^ with lorazepam being the most widely used.^1,3–5,12,43,44^ Lorazepam yields a response within minutes of intravenous administration at the usual dose of 1–2 mg. Fink and Taylor^1^ and Lucchini et al.^3^ showed a response rate of 50%–70% to a lorazepam challenge, while Pelzer et al.^36^ reported a 66%–100% response rate. Diazepam, clonazepam, and midazolam have also been effective.^44–48^ Once started, BZDs must be gradually withdrawn to avoid a recurrence of catatonia.^1,4,5,19,32,37^ A better response to BZDs has been described in catatonia associated with underlying affective disorders rather than that seen in schizophrenia.^24,32^

ECT is useful for severe cases or when there is no response to adequate BZDs treatment after 3 days.^1,4,5,18,20,24,49–52^ Furthermore, ECT is the treatment of choice for malignant catatonia (MC), a severe form of catatonia that presents with catalepsy (extreme muscle rigidity), fever, autonomic instability, and confusion.^1,4,5,36,49–52^ Lack of response to alternate-day administration of ECT or severe life-threatening catatonia may require daily ECT.^1,3–5,22,36^ ECT response rates of 80%–100% have been reported.^1,22,36,49–52^ Both unilateral and bilateral ECT are effective for catatonia, but show varying response rates, with bilateral ECT recommended in severe cases.^1,4,22,36^

Maintenance ECT may be indicated for recurring catatonia, with the frequency determined by patient need, from twice a week to once a month.^1,3,4,22,36,50^ Suzuki et al.^51^ examined the relapse rate after response to an acute ECT course administered during the active phase of catatonic schizophrenia, and found that the 1 year relapse rate tended to be high, with 57.1% of the participants experiencing relapse despite continuation of treatment with medication.

Anticonvulsants like carbamazepine, valproate, and topiramate may be alternative treatments for catatonia due to their GABA-related mechanism, which fits the GABA hypothesis of catatonia.^4,53^ Valproate has also been used for prophylaxis.^4,53^ Zolpidem also acts on the GABA system, and 10 mg may be used as an equivalent to the lorazepam challenge, three to four times a day.^4,54–56^

Other treatments that have been used include dopaminergic agents like bromocriptine, carbidopa, and l-dopa;^22,54^ *N*-methyl-d-aspartate receptor antagonists, like amantadine at doses of 100–600 mg/day, and memantine at doses of 10–20 mg/day;^4,54–56^ and atypical antipsychotics such as clozapine, olanzapine, risperidone, and quetiapine.^54,56^ Catatonia induced by clozapine withdrawal responds to clozapine re-initiation. Antipsychotics may, however, exacerbate the symptoms of catatonia or lead to neuroleptic malignant syndrome (NMS).^1,13,39^ White and Robins^39^ described NMS in 9 (53%) of 17 patients with catatonia after antipsychotic treatment, among whom 2 (11.8%) died. Other case studies indicate a mortality rate of 3%–5% with antipsychotics use, during an acute episode of catatonia.^39,56^ Fink and Taylor,^1^ Sienaert et al.,^4^ and Rosebush and Mazurek^42^ reported similar findings. This has led to the recommendation that antipsychotics should be avoided in cases of acute catatonia and should only be used when indicated or upon catatonia remittance.^1,4,39,42^

At the time of this study, the unit protocol consisted of an initial lorazepam challenge administered at a dose of 4 mg IV over 5 min. This dose is higher than that used in other protocols described elsewhere.^3–5,12,43–47^ The dose (2–4 mg) was repeated at 30 min intervals if catatonia showed no response. Lorazepam was continued (2–4 mg orally/IV) up to four times a day and adjusted according to the patient’s response. If the patient had prominent signs of withdrawal, stupor, or severe catatonia, lorazepam was administered intravenously every morning, with food, fluid, physiotherapy, and additional medication offered within an hour to maximize engagement and response.

Although there was no formal protocol to guide the administration of ECT, it tended to be administered to patients who were not taking food or water, those with autonomic instability, or those in severe catatonic excitement. Additional interventions involved multidisciplinary team members—nurses, a physiotherapist, dietician, occupational therapists, and specialists from other disciplines, such as neurologists, internists, and intensivists—to prevent further complications.^35^

Investigations are useful for identifying underlying causes of catatonia, monitoring treatment response,^57,58^ detecting decreased iron (Fe) levels^10,13,47,48,58–62^ and identifying increased creatinine kinase (CK) levels in severe catatonia.^10,13,47^

This study investigated the management of catatonia and treatment response in 44 patients during a 12-month prevalence study. We collected data on the management, investigations undertaken, treatment response, length of admission and outcomes at 4–8 weeks post-discharge, in patients with catatonia.

## Methods

This was a prospective, descriptive, observational study. The Bush Francis Catatonia Screening Instrument (BFCSI)/Bush Francis Catatonia Rating Scale (BFCRS), a predesigned data collection sheet and the *Diagnostic and Statistical Manual*-5 (DSM-5) were used to assess all new admissions to the unit from September 2020 to August 2021.^9,11^

### Site

The study site, an acute mental health unit (MHU), was a regional general hospital in Eastern Cape Province in South Africa, situated in the Nelson Mandela Metro, an urban city with 1.2 million people. Risk factors for mental health that the population in the Metro is exposed to include social determinants of health such as unemployment, poverty, and substance misuse.^63–67^ Mental health services at MHU include mental health assessments and ECT. Referrals to the MHU are received from other departments in the hospital as well as local clinics and district hospitals.

### Sampling

Convenience sampling was conducted for recruitment as part of a larger study on the prevalence of catatonia at the study site. The expected number of admissions during the study period was 1000. The margin of error, or CI, was set at 95% and the SD at 0.05. To determine the required total sample size, the formula n = N/(1 + Ne^2^) was applied. This yielded a minimum sample size of 286 participants, and another 20% (57, adding up to 343) were added in case of data entry errors. This number was slightly reduced to adjust for the influence of the COVID-19 pandemic on non-COVID-19-related causes of admission. Data collected from the case notes of participants included management and response to treatment during the inpatient period and recurrence of symptoms during one follow-up assessment in the first 4–8 weeks post-discharge.

### Inclusion and exclusion criteria

Those who screened positive for catatonia during the study period were recruited for participation. Participants who did not provide consent for recruitment into the study were excluded.

### Participants

The participants were mostly between the ages of 18 and 65 years with a mean age of 33.9, except for two who were older than 65 years.

### Study process

Recruitment and data collection were conducted by the researcher, supported by five research assistants (RAs) who were either psychiatry nurses or psychiatric residents working at the study site. All new admissions were assessed daily or within 48 h to accommodate weekdays and public holidays. In cases of catatonia, additional assessment information was shared with the treating physician. Two participants whose catatonia was initially missed were diagnosed by the research team over the 12-month study period, and we provided feedback to the treating team to allow for case review in those affected cases.

### Assessment tools

We used the BFCSI/BFCRS, DSM-5, and a data collection sheet to assess the participants for catatonia. Demographic and clinical information on the treatment, response, and outcomes were collected. BFCSI/BFCRS has been validated as a screening tool for catatonia, although it has not been validated in an African setting.^11^ The DSM-5 is a diagnostic tool used for all psychiatric disorders but has not been specifically validated for catatonia assessment.^9^ There is no validated tool for assessment of catatonia in the study population currently.

The inter-rater reliability (IRR) of the BFCSI/BFCRS was found to be good (α = 0.779) in one study that used four different instruments to assess for catatonia in India,^68^ as well as in another study conducted at the study site.^6^ The lead researcher provided training to the RAs on using the BFCSI/BFCRS and the data collection sheet, with piloting of the data sheet performed during the IRR assessment.^6^ The training covered the following areas:

- Meaning of terms or clinical signs and symptoms of catatonia.
- How to elicit and document the 14 items in the BFCSI and 23 items in the BFCRS, and how to capture the data accurately.

The IRR scores of the assessors during the assessment of the first 10 participants were good, as reflected in the study by Zingela et al.^6^

### Ethical considerations

Ethical approval for this study was obtained from the Nelson Mandela University Human Research Ethics Committee (Ref: H20-HEA-PSY-002).

The participants’ capacity to consent was assessed using a modified version of the University of California San Diego Brief Assessment of Capacity to Consent Questionnaire,^69^ and participants with the capacity to consent provided written consent. In the case of participants who lacked the capacity to provide consent, written proxy consent was obtained from legally authorized representatives before the study. This was permitted under the Mental Health Care Act of 2002.^70^

### Statistical analysis

All collected data was organized into categorical variables and presented as frequency tables and graphs. Logistic regression, Spearman’s rank correlations, and cross-tabulations were used to analyze the data and assess the associations between the prevalence of catatonia and interventions, blood tests, treatment outcomes, and clinical or demographic results.

## Results

### Treatment administered

Medications: Lorazepam, benzodiazepines, and other psychotropic medication: Up to 38 (86.4%) of the 44 participants with catatonia received a lorazepam challenge (IV administration to assess the immediate response) on admission. Seven (15.9%) participants received clonazepam and two (4.6%) received diazepam on admission. Thus, three participants received a combination of the three benzodiazepines at different times. This occurred when lorazepam was temporarily out of stock at the unit, with clinicians reverting to lorazepam as soon as the stock was replenished.

Responses to psychotropic medications were noted to be variable and did not follow a particular pattern, other than the worsening of catatonia with the addition of an antipsychotic, irrespective of the diagnosis. Those who worsened were mostly those who also showed low Fe levels on repeated checkups.

Thirty (68.2%) of the 44 participants received additional psychotropic medications (e.g. mood stabilizers, antipsychotics, or antidepressants) with lorazepam, clonazepam, diazepam, or ECT. None of the participants received first-generation antipsychotics during an acute episode of catatonia or an antipsychotic without a BZD.

### Electroconvulsive therapy

Ten (22.3%) of the 44 participants received ECT in addition to lorazepam. There was no specific indication in the clinical notes of why specific participants with catatonia were treated with ECT. However, from further extrapolation of the clinical notes, those who did not eat or drink fluids regularly or who were sometimes found “severely agitated” by the treating team were more likely to receive ECT.

Four (40%) of the 10 patients who received ECT were also on sodium valproate, and two (20%) were on clozapine, in addition to sodium valproate. While the BFCSI/BFCRS scores of both the group that received ECT and those that did not receive ECT were variable, the patients receiving ECT tended to show higher scores, with 8 (80%) of the 10 who received ECT having scores of 10 or above and only 2 who had scores below 10, specifically 2 and 8.

One of the 10 participants who required ECT had screened positive for catatonia on the BFCSI/BFCRS with a score of 2, but was not diagnosed with catatonia initially when using the DSM-5. The patient was subsequently diagnosed with catatonia based on further clinical assessment and a positive lorazepam challenge administered by the treating team on admission. This patient also required re-admission 4 weeks after discharge, owing to the recurrence of pronounced immobility and mutism.

The highest individual BFCRS score in the sample was 32 and was in the group that received ECT. Patients with higher BFCRS scores were more likely to be diagnosed with substance-induced psychotic disorders. The one participant with a BFCRS score of 32 was diagnosed with schizophrenia.

### Blood test results

All participants with catatonia and some without catatonic signs were tested for vitamin B12, serum iron, and CK levels at the time of admission. Among the 44 participants who screened positive for catatonia, 20 (45.5%) had low Fe levels, 14 (31.8%) had low vitamin B12 levels, and 24 (54.5%) had high CK levels. However, the number of participants in both the catatonia and non-catatonia groups was insufficient to draw additional conclusions from these findings.

Three participants with catatonia who were on lorazepam and an antipsychotic were sequentially tested for Fe levels a few days apart. Those with low Fe levels associated with catatonia who also received a co-administered antipsychotic initially showed further deterioration despite appropriate treatment with benzodiazepines. The Fe levels started to rise again, with progressive improvement in catatonic symptoms once the antipsychotic treatment was stopped. One of the three participants was eventually treated with ECT after the discontinuation of antipsychotic treatment did not lead to clinical improvement. The patient showed an improvement to the point of resolution of catatonia after treatment with ECT.

The others in the group with catatonia, whose iron levels were low, required no iron supplementation because their iron levels normalized when the catatonia improved with treatment. However, those with low vitamin B12 levels required supplementation as no improvement was observed in their vitamin B12 levels, even after catatonic symptoms responded to the treatment.

### Vital signs (blood pressure, pulse, temperature, and oxygen saturation)

The blood pressure, pulse, and temperature results of 31 (70.5%) participants; the oxygen saturation results of 30 (68.2%) participants; and the respiratory rate of 8 (18.2%) participants from among the 44 with catatonia were recorded. All parameters that were taken at the point of admission and before treatment for catatonia were administered. The findings are summarized in Table 1.

**Table 1.** Vital signs.

**CROSS-TABULATIONS**								
			**Systolic Blood Pressure**					Total
			Not answered	<120		120–139	140–180	
**BFCRS Diagnosis**	Not Catatonic	Count	192	2		2	1	197
		% of Total	79.7%	0.80%		0.80%	0.4%	81.70%
	Catatonic	Count	13	20		11	0	44
		% of Total	5.4%	8.3%		4.6%	0.0%	18.3%
**Total**		Count	205	22		13	1	241
		% of Total	85.1%	9.1%		5.4%	0.4%	100.0%
Cross-tabulations								
			**Diastolic Blood Pressure**					Total
			Not answered	<70	80–90	91–110	110–120	
**BFCRS Diagnosis**	Not Catatonic	Count	192	4	0	0	1	197
		% of Total	79.7%	1.7%	0.0%	0.0%	0.4%	81.7%
	Catatonic	Count	13	19	10	2	0	44
		% of Total	5.4%	7.9%	4.1%	0.8%	0.0%	18.3%
**Total**		Count	205	23	10	2	1	241
		% of Total	85.1%	9.5%	4.1%	0.8%	0.4%	100.0%

A number of patients with catatonia had a systolic blood pressure (SBP) of less than 120 mm Hg (20; 45.5%) and a diastolic blood pressure (DBP) of less than 70 mm Hg (19; 43.2%). The pulse levels of 20 (45.5%) of the 44 participants were in the range of 70–100 beats per minute, and 31 (70.5%) of the participants had temperature readings between 35°C and 37°C. The oxygen levels of 30 (68.2%) participants were available, of whom 8 had catatonia and 24 (54.5%) had levels between 97% and 99%. Table 1 shows the SBP and DBP of the participants.

### Complications of Catatonia

None of the participants had any major complications of catatonia during either admission or monitoring during the follow-up period.

### Response to treatment

Of the 44 participants with catatonia, 14 (31.9%) were admitted at the end of the 12-month study period, while 29 (65.9%) had been discharged. One (2.3%) of the 44 patients was referred to a local psychiatric hospital for further management. Another patient (2.3%) presented with a recurrence of severe motor slowing, rigidity, and mutism after discharge. They were on risperidone without BZDs for 8 weeks prior to re-admission. The treating team was alerted by the research team regarding the recurrence of catatonia. The patient was re-admitted, restarted on lorazepam, and risperidone was discontinued.

Among the 10 patients with catatonia who received ECT, 7 (15.9% of the 44) were discharged, while 3 (6.8% of the 44) were still admitted. This means that 22 (50%) of those discharged had received lorazepam without ECT. Additional psychotropic medications used in combination with lorazepam include sodium valproate and clozapine.

### Length of admission

The average length of admission for 30 (68.2%) of the 44 participants with catatonia who were discharged by the end of the study period was 66.5 days, while the average length of stay for those without catatonia for the same study period was 65.3 days. This implies that the length of admission in the majority of the 44 patients with catatonia was similar to those without catatonia. Table 2 summarizes the clinical and demographic parameters of participants with catatonia.

**Table 2.** Clinical and demographic parameters.

Clinical/demographic info	No. of participants	Minimum	Maximum	Mean	Std. deviation
Age	44	17	65	31.6	11.49
Length of stay (days)	39	9	290	84.9	54.36
B12 level	43	0	545	53.7	130.90
Ck value	36	32	1703	289.2	346.52
Fe value	35	1.3	33.7	12.7	7.55

### Follow-up period

One patient (2.3%) was re-admitted during the study period. The remaining 29 (65.9% of 44) participants showed no symptoms at the 4- and 8-week follow-up points, with no recurrence of catatonia in BFCSI/BFCRS.

### Analysis of results

The analysis consisted of cross-tabulations to determine the association between the BFCSI/BFCRS scores. BFCSI/BFCRS was used in this analysis because it identified the highest number of catatonia cases that screened positive, and Table 3 shows several significant associations.

**Table 3.** Cross-tabulations of vital signs.

Value	χ^2^	Sig.	*v*
Systolic	135.893	0.000	0.751
Diastolic	137.275	0.000	0.755
Pulse	135.739	0.000	0.750
Temperature	135.65	0.000	0.750
Oxygen saturation	150.575	0.000	0.792

A statistically significant association was found between positive BFCRS scores and SBP (χ^2^ (3) = 135.893, sig. = 0.000, ν = 0.751). A significant percentage of patients with catatonia had an SBP < 120 mm Hg (n = 20). This was followed by 11 patients with SBP of between 120 and 139 mm Hg.

A statistically significant association was also found between positive BFCRS and DBP (χ^2^ (4) = 137.275, sig. = 0.000, ν = 0.755). A significant percentage of patients with catatonia had a DBP of <70 mm Hg (n = 19). This was followed by 10 patients with a DBP of 80–90 mm Hg.

A statistically significant association was found between positive BFCRS and pulse rate (χ^2^ (4) = 135.739, sig. = 0.000, ν = 0.750). A significant percentage of patients with catatonia had pulse rates of between 71 and 100 beats per minute (n = 20). This was followed by five with pulse rates below 70, and four with pulse rates between 101 and 120 beats per minute.

A statistically significant association was found between the BFCRS and temperature (χ^2^ (1) = 135.65, sig. = 0.000, ν = 0.750). A significant percentage of patients with catatonia had temperatures between 35°C and 37°C (n = 31).

A statistically significant association was also found between the BFCRS and oxygen saturation levels (χ^2^ (2) = 150.575, sig. = 0.000, ν = 0.792). A significant percentage of the patients with catatonia had oxygen saturation levels of between 97% and 99% (n = 24).

### Summary of findings

This study yielded the following significant findings: close to 23% of patients required ECT in addition to benzodiazepine treatment. There is no firmly established unit protocol to guide clinicians on who should be treated with ECT when presenting with catatonia. Those who received ECT for catatonia during the study period had a higher BFCSI/BFCRS score, poor to no oral intake of fluids or food, catatonic excitement, and autonomic instability. This lack of guidelines or protocols for ECT poses an additional limitation to the generalizability of the study findings.

Outcomes were generally good in the short-term, with discharge from the hospital after an average stay of 8–12 weeks. The relapse rate was 2.3%, and the one patient who relapsed was re-admitted within 4 weeks of discharge. Up to 70% of the patients treated with ECT showed a good response and were discharged on oral treatment, which included lorazepam to avoid abrupt withdrawal. The mean values for iron (12.7 µg/L) and vitamin B12 (153.74 pmol/L) tended to be on the lower side, with high values for creatine kinase (289.1 U/L).^53,71^

Blood pressure, pulse, and temperature tended to be normal in those who screened positive for catatonia in the sample, although a subset showed lower SBP (20; 45.5%) and DBP (19; 43.2%) than normal, which is an indication of the severity of catatonia according to the BFCRS items. One patient screened positive for catatonia with a BFCSI score of 2 plus a response to a lorazepam challenge. However, the response was not sustained, and the symptoms were significant enough to warrant treatment with ECT.

## Discussion

This study presented data on the treatment administered, additional clinical parameters such as vital signs (blood pressure, pulse, temperature, and oxygen saturation), and course of illness in 44 of 241 participants who were found to have catatonia during the 12-month screening period.

The results showed that catatonia responded well to lorazepam, and ECT was administered to those with more severe catatonic symptoms in this cohort. The fact that 10 (22.7% of 44) participants who received ECT tended to have higher BFCRS scores implies that severity was the underlying determinant of ECT administration in this sample.

The length of admission and recurrence of catatonia during the first 4–8 weeks following discharge also confirmed the good outcomes of catatonia management at the study site. Those whose catatonia responded to treatment spent approximately the same amount of time in the hospital as patients without catatonia. None of the patients had complications of catatonia during the period of admission or 4–8 weeks post-discharge. This further supports the conclusion that in this cohort, the outcomes of catatonia were generally good in most patients, with no fatalities.

The clinical implications of the findings in this section are that lorazepam and ECT in patients with severe catatonia or high BFCRS are good interventions that yield good outcomes. The findings of low blood pressure imply the need to monitor this in patients with catatonia. Finally, low iron levels in patients with catatonia were also demonstrated in a recent study,^57^ highlighting the need to monitor iron levels.

Issues of consent for ECT during an episode of catatonia may pose an ethical challenge because affected patients may be unresponsive and uncommunicative. In such instances, it is important to balance risk to life against the risk of infringement of rights in someone who is unable to indicate assent or dissent while in an acute state of catatonia. The Mental Health Care Act was used in the current study to assist clinicians in navigating these ethical challenges. The use of proxy consent was also appropriately instituted for patients with catatonia at the study site and its application supported protection of the patient’s rights during the recruitment and sampling process. Those who improved were presented with the opportunity to reconsent. None of the patients with catatonia refused ECT once their ability to consent was restored. This further indicates that it is possible to conduct ethical research on patients with catatonia, even during acute catatonia. This is highlighted because research on patients with catatonia in South Africa has been mainly retrospective, with consent obtained from institutional managers to gain access to the clinical records of patients with catatonia.

### Strengths

This is one of the very few studies on catatonia in South Africa and provides preliminary findings that may contribute to future research on catatonia in the South African and African contexts. Findings on the relationship between catatonia, iron levels, and vitamin B12 indicate a need for further studies to clarify the potential clinical implications.^54^ This could include the potential use of parameters such as iron and CK levels for monitoring the clinical response to treatment of catatonia. This is based on the fact that a subset of patients with catatonia in this cohort showed a change in iron and CK levels, with changes in their clinical status, treatment, and severity of catatonia.

### Limitations

The COVID-19 pandemic affected the recruitment rates. Previous projections on the number required for recruitment had to be revised to less than 40 participants, with actual recruitment ending up slightly better than anticipated. The limited number of available blood results, mostly once-off assessments, and lack of a formalized treatment protocol for the initiation of ECT indicated the variability of treatment offered to patients with catatonia and posed a limitation to the interpretation of results. This affected the ability to collect data from what would have otherwise been a more homogeneous subgroup, as potentially those who received ECT had a standardized ECT treatment protocol. This may have enabled a more in-depth assessment of those who were more likely to require ECT for catatonia.

The BFCSI/BFCRS and DSM-5 have not been validated in the study population, which is a limitation. The piloting of the assessment tools and inclusion of an inter-rater reliability assessment in the study process,^6^ were used as potential mitigation for such limitations.

Future studies should consider the role of serial iron levels before and during treatment in patients with catatonia. The relationship between vitamin B12, cannabis, alcohol use, and catatonia also require further investigation. Furthermore, determining the most cost-effective treatment for catatonia considering the limited resources in South Africa could make clinicians more selective in their treatment choices. Access to ECT services and clinician choice might also explain why many patients with catatonia in this subset were treated with multiple psychotropic medications over and above the standard treatment of catatonia, with lorazepam and ECT.

## Conclusion

Lorazepam and ECT are the most widely used and effective treatments for catatonia. The treatment response and outcomes were good, and supportive treatment was beneficial. The length of hospital stays for patients with catatonia was similar to that of patients without catatonia. Treatment guidelines for catatonia, with a specific focus on the role and timing of ECT need to be developed and added to the current protocols for acute settings to augment the current treatment protocols for the use of lorazepam. Once the subjective aspects of catatonia are better understood, treatment guidelines could include psychological (e.g. supportive, cognitive, and behavioral) strategies, especially when subjective experiences are found to reflect significant distress or suffering.

## Supplemental Material

sj-docx-1-smo-10.1177_20503121221105579 – Supplemental material for Management and outcomes of catatonia: A prospective study in urban South Africa Click here for additional data file. Supplemental material, sj-docx-1-smo-10.1177_20503121221105579 for Management and outcomes of catatonia: A prospective study in urban South Africa by Zukiswa Zingela, Louise Stroud, Johan Cronje, Max Fink and Stephan van Wyk in SAGE Open Medicine

## References

[1] Fink M Taylor MA . Catatonia: a clinician’s guide to diagnosis and treatment. Cambridge: Cambridge University Press, 2003, pp. 15–30.

[2] Kaelle J Abujam A Ediriweera H , et al. Prevalence and symptomatology of catatonia in elderly patients referred to a consultation-liaison psychiatry service. Australas Psychiatry 2016; 24(2): 164–167. 26400451 10.1177/1039856215604998

[3] Luchini F Medda P Mariani MG , et al. Electroconvulsive therapy in catatonic patients: efficacy and predictors of response. World J Psychiatry 2015; 5(2): 182–192. 26110120 10.5498/wjp.v5.i2.182 PMC4473490

[4] Sienaert P Dhossche DM Vancampfort D , et al. A clinical review of the treatment of catatonia. Front Psychiatry 2014; 5: 181. 25538636 10.3389/fpsyt.2014.00181 PMC4260674

[5] Solmi M Pigato GG Roiter B , et al. Prevalence of catatonia and its moderators in clinical samples: results from a meta-analysis and meta-regression analysis. Schizophr Bull 2018; 44(5): 1133–1150. 29140521 10.1093/schbul/sbx157 PMC6101628

[6] Zingela Z Stroud L Cronje J , et al. Assessment of catatonia and inter-rater reliability of three instruments: a descriptive study. Int J Ment Health Syst 2021; 15(1): 82. 34809692 10.1186/s13033-021-00505-8 PMC8607401

[7] Zingela Z Stroud L Cronje J , et al. A prospective descriptive study on prevalence of catatonia and correlates in an acute mental health unit in Nelson Mandela Bay, South Africa. PLoS ONE 2022; 17(3): e0264944. 10.1371/journal.pone.0264944 PMC8903294 35259194

[8] Zingela Z Stroud L Cronje J , et al. The psychological and subjective experience of catatonia [Preprint] (Version \#) available at Research Square 2022, https://www.researchsquare.com/article/rs-1420539/v1 10.1186/s40359-022-00885-7 PMC9287913 35841077

[9] American Psychiatric Association. Anxiety disorders. In: Diagnostic and statistical manual of mental disorders. 5th ed. 2013. pp. 119–120, https://dsm.psychiatryonline.org/doi/10.1176/appi.books.9780890425596.dsm05

[10] Bhati MT Datto CJ O‘Reardon JP . Clinical manifestations, diagnosis, and empirical treatments for catatonia. Psychiatry 2007; 4(3): 46–52. PMC2922358 20805910

[11] Bush G Fink M Petrides G , et al. Catatonia: I—rating scale and standardized examination. Acta Psychiatr Scand 1996; 93(2): 129–136 8686483 10.1111/j.1600-0447.1996.tb09814.x

[12] Fink M Shorter E Taylor MA . Catatonia is not schizophrenia: Kraepelin’s error and the need to recognize catatonia as an independent syndrome in medical nomenclature. Schizophr Bull 2010; 36(2): 314–320. 19586994 10.1093/schbul/sbp059 PMC2833121

[13] Rasmussen SA Mazurek MF Rosebush PI . Catatonia: our current understanding of its diagnosis, treatment and pathophysiology. World J Psychiatry 2016; 6(4): 391–398. 28078203 10.5498/wjp.v6.i4.391 PMC5183991

[14] World Health Organization. International classification of diseases for mortality and morbidity statistics. 11th Revision. Geneva: World Health Organization, 2018.

[15] Tandon R Heckers S Bustillo J , et al. Catatonia in DSM-5. Schizophr Res 2013; 150(1): 26–30. 23806583 10.1016/j.schres.2013.04.034

[16] Burrow JP Spurling BC Marwaha R. Catatonia . In: StatPearls. Treasure Island, FL: Stat Pearls Publishing, 2020.

[17] Caroff SN Mann SC Francis A , et al. Catatonia from psychopathology to neurobiology. Washington, DC: American Psychiatric Publishing. 2007, pp. 45–52.

[18] Carrol BT Pinson V . Catatonia: diagnostic approaches and therapeutic management. Future Neurol 2015; 10(5): 393–394.

[19] Daniels J . Catatonia: clinical aspects and neurobiological correlates. J Neuropsychiatry Clin Neurosci 2009; 21(4): 371–380. 19996245 10.1176/jnp.2009.21.4.371

[20] Edinoff AN Kaufman SE Hollier JW , et al. Catatonia: clinical overview of the diagnosis, treatment, and clinical challenges. Neurol Int 2021; 13(4): 570–586. 34842777 10.3390/neurolint13040057 PMC8628989

[21] Wilson JE Niu K Nicolson SE , et al. The diagnostic criteria and structure of catatonia. Schizophr Res 2015; 164(1–3): 256–262. 25595653 10.1016/j.schres.2014.12.036

[22] Smith SL Grelotti DJ Fils-Aime R , et al. Catatonia in resource-limited settings: a case series and treatment protocol. Gen Hosp Psychiatry 2015; 37(1): 89–93. 25467078 10.1016/j.genhosppsych.2014.10.009 PMC4304795

[23] Wong JW Williams SR . The wandering woman: a case study of catatonia vs factitious disorder. Hawaii J Med Public Health 2017; 76(3): 82–84. 28352494 PMC5349116

[24] Francis A . Catatonia: diagnosis, classification, and treatment. Curr Psychiatry Rep 2010; 12(3): 180–185. 20425278 10.1007/s11920-010-0113-y

[25] Mormando C Francis A . Catatonia revived: a unique syndrome updated. Int Rev Psychiatry 2020; 32(5–6): 403–411. 32067538 10.1080/09540261.2020.1723500

[26] Novac AA Bota D Witkowski J , et al. Special medical conditions associated with catatonia in the internal medicine setting: hyponatremia-inducing psychosis and subsequent catatonia. Perm J 2014; 18(3): 78–81. 10.7812/TPP/13-143 PMC4116269 25102520

[27] Pommepuy N Januel D . La catatonie: résurgence d’un concept—Une revue de la littérature internationale [Catatonia: resurgence of a concept—a review of the international literature]. Encephale 2002; 28(6 Pt. 1): 481–492. 12506260

[28] Northoff G . Catatonia and neuroleptic malignant syndrome: psychopathology and pathophysiology. J Neural Transm 2002; 109(12): 1453–1467. 12486486 10.1007/s00702-002-0762-z

[29] Iyengar S Bornmann C Abdelmalak F , et al. Catatonia due to alprazolam withdrawal. BMJ Case Rep 2018; 11(1): e227175. 10.1136/bcr-2018-227175 PMC6301493 30567266

[30] Northoff G Krill W Wenke J , et al. The subjective experience in catatonia: systematic study of 24 catatonic patients. Psychiatr Prax 1996; 23(2): 69–73. 8657812

[31] Shorter E Fink M . The madness of fear: A history of catatonia. Oxford: Oxford University Press, 2018, pp. 79–92.

[32] Espinola-Nadurille M Flores-Rivera J Rivas-Alonso V , et al. Catatonia in patients with anti-NMDA receptor encephalitis. Psychiatry Clin Neurosci 2019; 73(9): 574–580. 31115962 10.1111/pcn.12867

[33] Mythri SV Mathew V . Catatonic syndrome in anti-NMDA receptor encephalitis. Indian J Psychol Med 2016; 38(2): 152–154. 27114630 10.4103/0253-7176.178812 PMC4820557

[34] McCall WV Mann SC Shelp FE , et al. Fatal pulmonary embolism in the catatonic syndrome: two case reports and a literature review. J Clin Psychiatry 1995; 56(1): 21–25. 7836335

[35] Gross AF Smith FA Stern TA . Dread complications of catatonia: a case discussion and review of the literature. Prim Care Companion J Clin Psychiatry 2008; 10(2): 153–155. 18458717 10.4088/pcc.v10n0210 PMC2292429

[36] Pelzer AC van der Heijden FM den Boer E . Systematic review of catatonia treatment. Neuropsychiatr Dis Treat 2018; 14: 317–326. 29398916 10.2147/NDT.S147897 PMC5775747

[37] Grover S Ghosh A Ghormode D . Do patients of delirium have catatonic features? An exploratory study. Psychiatry Clin Neurosci 2014; 68(8): 644–651. 24521083 10.1111/pcn.12168

[38] Stuivenga M Morrens M . Prevalence of the catatonic syndrome in an acute inpatient sample. Front Psychiatry 2014; 5: 174. 25520674 10.3389/fpsyt.2014.00174 PMC4253531

[39] White DA Robins AH . An analysis of 17 catatonic patients diagnosed with neuroleptic malignant syndrome. CNS Spectr 2000; 5(7): 58–65. 10.1017/s1092852900013419 18197157

[40] Lloyd JR Silverman ER Kugler JL , et al. Electroconvulsive therapy for patients with catatonia: current perspectives. Neuropsychiatr Dis Treat 2020; 16: 2191–2208. 33061390 10.2147/NDT.S231573 PMC7526008

[41] Fricchione GL Cassem NH Hooberman D , et al. Intravenous lorazepam in neuroleptic-induced catatonia. J Clin Psychopharmacol 1983; 3(6): 338–342. 6139391

[42] Rosebush PI Mazurek MF . Catatonia and its treatment. Schizophr Bull 2010; 36(2): 239–242. 19969591 10.1093/schbul/sbp141 PMC2833127

[43] Suchandra HH Reddi VSK Aandi Subramaniyam B , et al. Revisiting lorazepam challenge test: clinical response with dose variations and utility for catatonia in a psychiatric emergency setting. Australian & New Zealand Journal of Psychiatry 2021; 55(10): 993–1004. 33124447 10.1177/0004867420968915

[44] Wick JY . The history of benzodiazepines. Consult Pharm 2013; 28(9): 538–548. 24007886 10.4140/TCP.n.2013.538

[45] Jiang S Brownell KC Kamper JE , et al. Clonazepam for catatonia incompletely responsive to lorazepam. J Acad Consult Liaison Psychiatry 2021; 62(1): 97–101. 33268101 10.1016/j.psym.2020.09.009

[46] Oğuz S Tuygun N Kesici S , et al. Successful treatment of unknown drug-induced catatonia with intravenous midazolam in a nine-year-old girl. Bezmiâlem Sci 2018; 6(1): 70–72.

[47] Rosebush PI Hildebrand AM Furlong BG , et al. Catatonic syndrome in a general psychiatric inpatient population: frequency, clinical presentation, and response to lorazepam. J Clin Psychiatry 1990; 51(9): 357–362. 2211547

[48] Mustafa FA . Intravenous Midazolam as a diagnostic test for Catatonia. J ECT 2017; 33(4): e36. 10.1097/YCT.0000000000000439 28658008

[49] Singh A Kar SK . How electroconvulsive therapy works? Understanding the neurobiological mechanisms. Clin Psychopharmacol Neurosci 2017; 15(3): 210–221. 28783929 10.9758/cpn.2017.15.3.210 PMC5565084

[50] Fink M Taylor MA . Electroconvulsive therapy: evidence and challenges. JAMA 2007; 298(3): 330–332. 17635894 10.1001/jama.298.3.330

[51] Suzuki K Awata S Takano T , et al. Continuation electroconvulsive therapy for relapse prevention in middle-aged and elderly patients with intractable catatonic schizophrenia. Psychiatry Clin Neurosci 2005; 59(4): 481–489. 16048455 10.1111/j.1440-1819.2005.01402.x

[52] Park J Tan J Krzeminski S , et al. Malignant catatonia warrants early psychiatric-critical care collaborative management: two cases and literature review. Case Rep Crit Care 2017; 2017: 1951965. 28250995 10.1155/2017/1951965 PMC5303832

[53] Kritzinger PR Jordaan GP . Catatonia: an open prospective series with carbamazepine. Int J Neuropsychopharmacol 2001; 4(3): 251–257. 11602030 10.1017/S1461145701002486

[54] Beach SR Gomez-Bernal F Huffman JC , et al. Alternative treatment strategies for catatonia: a systematic review. Gen Hosp Psychiatry 2017; 48: 1–19. 28917389 10.1016/j.genhosppsych.2017.06.011

[55] Peglow S Prem V McDaniel W . Treatment of catatonia with zolpidem. J Neuropsychiatry Clin Neurosci 2013; 25(3): E13. 10.1176/appi.neuropsych.11120367 24026726

[56] Yoshimura B Hirota T Takaki M , et al. Is quetiapine suitable for treatment of acute schizophrenia with catatonic stupor? A case series of 39 patients. Neuropsychiatr Dis Treat 2013; 9: 1565–1571. 24143105 10.2147/NDT.S52311 PMC3797635

[57] Brasic JR Farhadi F . Catatonia workup. Medscape drugs and diseases: neurology, 2018, https://emedicine.medscape.com/article/1154851-workup (accessed 18 November 2021).

[58] Peralta V Cuesta MJ Mata I , et al. Serum iron in catatonic and noncatatonic psychotic patients. Biol Psychiatry 1999; 45(6): 788–790. 10188010 10.1016/s0006-3223(98)00137-1

[59] Fink M Taylor MA . The many varieties of catatonia. Eur Arch Psychiatry Clin Neurosci 2001; 251(1 Suppl. 1): I8–II13. 10.1007/pl00014200 11776271

[60] Rogers JP Pollak TA Begum N , et al. Catatonia: demographic, clinical and laboratory associations. Psychol Med. Epub ahead of print 2 November 2021. DOI: 10.1017/S0033291721004402. PMC10123832 35135642

[61] Lee JW . Serum iron in catatonia and neuroleptic malignant syndrome. Biol Psychiatry 1998; 44(6): 499–507. 9777183 10.1016/s0006-3223(98)00109-7

[62] Phatlhane DV Zemlin AE Matsha TE , et al. The iron status of a healthy South African adult population. Clin Chim Acta 2016; 460: 240–245. 27339094 10.1016/j.cca.2016.06.019

[63] Lund C Breen A Flisher AJ , et al. Poverty and common mental disorders in low and middle income countries: a systematic review. Soc Sci Med 2010; 71(3): 517–528. 20621748 10.1016/j.socscimed.2010.04.027 PMC4991761

[64] Thungana Y Zingela Z van Wyk S . First-episode psychosis and substance use in Nelson Mandela Bay: findings from an acute mental health unit. S Afr J Psychiatr 2019; 25(0): 1372–1376. 31745443 10.4102/sajpsychiatry.v25i0.1372 PMC6851873

[65] World Health Organization. Social determinants of mental health. Geneva: World Health Organization, 2014, pp. 36–38, https://apps.who.int/iris/bitstream/handle/10665/112828/9789241506809_eng.pdf, (accessed 5 January 2022).

[66] Statistics South Africa. Statistics South Africa to release the Quarterly Labour Force Survey (QLFS), 2nd Quarter 2019, 2019. Republic of South Africa, http://www.statssa.gov.za/?p=12358 (accessed 24 March 2021).

[67] Statistics South Africa: 2020. Nelson Mandela Bay. Republic of South Africa, http://www.statssa.gov.za/?page_id=1021&id=nelson-mandela-bay-municipality (2020, accessed 25 October 2020).

[68] Sarkar S Sakey S Mathan K , et al. Assessing catatonia using four different instruments: inter-rater reliability and prevalence in inpatient clinical population. Asian J Psychiatr 2016; 23: 27–31. 27969074 10.1016/j.ajp.2016.07.003

[69] Campbell MM Susser E Mall S , et al. Using iterative learning to improve understanding during the informed consent process in a South African psychiatric genomics study. PLoS One 2017; 12(11): e0188466. 10.1371/journal.pone.0188466 PMC5707000 29186155

[70] Mental Health Care Act. (Act no. 17 of 2002) General Regulations [Internet].

[71] Berg RL Shaw GR . Laboratory evaluation for vitamin B12 deficiency: the case for cascade testing. Clin Med Res 2013; 11(1): 7–15. 23262189 10.3121/cmr.2012.1112 PMC3573090

