# Supplementary material for: Management and outcomes of catatonia: A prospective study in urban South Africa
Source: SAGE Open Med. 2022 Jun 20;10:20503121221105579. doi: 10.1177/20503121221105579 (PMC9218450; doi:10.1177/20503121221105579)
Supplement: sj-docx-1-smo-10.1177_20503121221105579 – Supplemental material for Management and outcomes of catatonia: A prospective study in urban South Africa [file sj-docx-1-smo-10.1177_20503121221105579.docx]

**APPENDIX A: DATA COLLECTION SHEET**

| **Data Pack No:** | | | | **Tick applicable box or insert answer in area shaded in white** | | | | | | | | | | |
| --- | --- | --- | --- | --- | --- | --- | --- | --- | --- | --- | --- | --- | --- | --- |
| **Unit** | | | | **Is the Patient Catatonic now?**  **(Fill in the BFCRS item 1 to 14 to answer this question), 2 or more signs mean Yes, there is catatonia** | | | | | | | | | | |
| DNH | | | | If No then tick this box and fill in ONLY Sections A,G,H,I, and J | | | | | | | If Yes then tick this box and fill in sections A, B, C, D, E, F, G, H, I, and J. | | | |
| **A. Age** | | | | **Sex** | | **Ethnicity** | | | | **1st admission** | **Catatonic symptoms before?** | **Provisional DSM -5**  **Diagnosis**  **in file?** | **Susbtance Use?** | **Another medical condition** |
| < 16 | 6 -  35 | 36 - 65  5 | >  65 | F (0) | M (1) | BB | CC | WI | WW | Yes  No | Yes  No | Yes  Diagnosis  ……………….  No | Yes  No | Yes  No |
|  |  |  |  |  |  |  |  |  |  | Admissions previously?  0  1  >1 | Catatonic previously?  0  1  >1 | No of previous diagnoses?  0  1  >1 | For how Long?  < 4 Wks  1-3 Mths  >6 Mths | Medical conditions?  0  1  >1 |
|  |  |  |  |  |  |  |  |  |  | Not known | Not known | Not known | Not known | Not known |
| **B.**  **BFCR Score and other clinical parameters.**  For BFCR Score, fill in the White Box below according to response before and after BZD dose | | **BZD or Given?**  **Y / N?**  **If Yes, Dose?**  1-2mg  2-4 mg  5 -10 mg  >10mg  **No of dose/s**  1 -2  3 - 4  5 or more | | **Name of BZD given:**  **BZD or Rx given:**  Lorazepam  Clonazepam  Diazepam  Midazolam  Other Treatment?  1.Antipsychotic  2. Anticonvulsant  3.Lithium  4. Antidepressant  5. Clexane  6. Other | | | | **Blood Pressure** | | | **Pulse**  <70  71-100  101-120  121-160  >160 | **Body Temp**  <35  35 -37  38 – 40  >40 | **Respiratory Rate/ O2 Sats:**  <90%  91 – 93  94 – 96  97 – 99  100  **RATE:**  <8  8-16  17-26  26-30  >30 | |
|  |  |  |  |  |  |  |  | **Systolic:**  <120  120 -139  140 – 180  181 – 220  >220 | | **Diastolic:**  <70  80 -90  91-110  110-120  >120 |  |  |  |  |
| Initial Score before BZD administration  <6  6-12  12-24  24-36  >36 | | | | After 1^st^ BZD dose  <6  6-12  12-24  24-36  >36 | | After 2^nd^ BZD dose  <6  6-12  12-24  24-36  >36 | | | | After 3^rd^ BZD dose  <6  6-12  12-24  24-36  >36 | | After 4^th^ BZD dose  <6  6-12  12-24  24-36  >36 | After 5^th^ BZD dose  <6  6-12  12-24  24-36  >36 | |

| **C. Length of time and degree of response to BZD** | 1^st^ hour after admission | | 2-3 days | | | 4-6 days | | | **Degree of Response** | | | |
| --- | --- | --- | --- | --- | --- | --- | --- | --- | --- | --- | --- | --- |
|  |  |  |  |  |  |  |  |  | Mild = Less than 25% reduction in No. of symptoms | | | |
|  | 2 to 6 hours after admission | | 7-10 days | 11-14 days | | >14 days | | | Moderate = 25% to 50% reduction in No. of symptoms | | | |
|  | 7 to 47 hours after admission | | Reason ECT was not given after the 1^st^ 3 days of admission (from clinical notes)?  Reason Stated in File?: Y / N  If Yes, stated in file, write reason below: ……………………………………………….  ………………………………………………. | | | | | | Good = Response of more than 50% reduction in No. of symptoms | | | |
|  |  |  |  |  |  |  |  |  | Response to BZD not sustained | | | |
| **D.**  **ECT and response** | Yes | | **Number of**  **Sessions** | **Response** | | | | | Response to ECT not sustained | | | |
|  |  |  | <4  5-9  10-12  >12 | Nil  Remission of catatonia  Other (specify)……………...  ……………………………... | | | | | **Maintenance ECT prescribed or required?** | | | |
|  | No | |  | Time to 50% improvement  <3 days  4-7 days  >1 week | | Time to full Resolution  <3 days  4-7 days  >1 week | | | Yes, prescribed?  If so what is the No. of sessions? | | No, not prescribed | |
| **E.**  **Duration of Catatonia prior to admission if known**  OR tick here if …………  **Not Known** | Hours to 3  days | **Duration of Catatonia Prior to admission?** | Any other additional information? | | | | | | | | | |
|  | 4 days  to 2  weeks | < 3 days  4 to 7 days  >7 days  OR Tick here if  Not Known |  |  |  |  |  |  |  |  |  |  |
|  | 3 to 4  weeks |  |  |  |  |  |  |  |  |  |  |  |
|  | More than 4  weeks |  |  |  |  |  |  |  |  |  |  |  |
| **F. Type of onset** | Hours to  days | Gradual | Fluctuating | **Mostly Excited Form?** | | | | **Mostly Slowed Form?** | | | | |
|  |  |  |  | Excited/ Stereotypy/ Mannerism | | | | Stupor/ Withdrawal/ Rigidity/ Mutism/ Staring | | | | |
| **G.**  **Food Insecurity** | 1.Within the past 12 months, we worried whether our food would run out before we got money to buy more. | | | | | | | | | | | |
|  | Often  True | Sometimes  true | Never true | Don’t know | | Other | | | | | | |
|  | 2.Within the past 12 months, the food we bought just didn’t last and we didn’t have money to get more. | | | | | | | | | | | |
|  | Often  true | Sometimes  true | Never true | Don’t know | | Other | | | | | | |
| **H.**  **Substances** | | YES. How Often/ week?  1-3  4-6  6-7  >7 | NO | Alcohol | Cannabis | | Amphet | | | Heroin | | Metamphet |
|  |  |  |  | Cocaine | Opiods | | Nicotine | | | Other (Specify) | | |

| **I.Medical Illness** | No | Yes | If Yes, choose from the following if on history only | | If Yes, choose from the following if current | | | If HIV | | | |
| --- | --- | --- | --- | --- | --- | --- | --- | --- | --- | --- | --- |
|  |  |  | HPT | DM | HPT | DM | | On HAART? | | No | |
|  |  |  | Epilepsy | HIV | Epilepsy | HIV | | If HIV on HAART,  Regime?  …………………………………… | | If HIV, CD4?  >200  201-500  501 -1000  >1000 | |
|  |  |  | Head Trauma | TB | Head  Trauma | TB | | If HIV, Viral Load?  Undetectable  Detectable | | Other (specify) | |
|  |  |  | SLE or Auto/I | Other (specify) | | Other (specify) | | | |  |  |
| **J.**  **Investigations:**  **1.CK – Creatinine Kinase**  **2.Fe – Iron**  **3.B12 – Vitamin B12**  **4.TSH – Thyroid Stimulating Hormone**  **5.T4 – Thyroid Hormone**  **6.ANF – Nuclear Factor**  **Rheumatoid Factor** | **CK (u/l)**  **Highest Value** | | CK≤200 | **Fe µmol/l**  **Lowest Value** | Fe 9 to 30 | **VitB12**  **pmol/l** | | B12 ≤ 107 | | **Auto/I**  **Screen** | |
|  | CK  201 | -1000 | CK  ≥1000 | Fe  ≤ 9 | Fe  ≥ 30 | B12  108 - 221 | | B12  ≥ 221 | RF | ESR | ANA |
|  |  |  |  |  |  |  |  |  | < 14IU/  ml  OR  >14IU/  ml | < 29  OR  > 29  A | Pos OR  Neg |
|  |  |  |  | Fe  >9 to 29 |  |  |  |  |  |  |  |
|  | **Endocrine** | | **TSH miu/l** | TSH  ≤0.38 | TSH  ≥5.33 | **Cortisol** | | Cortisol AM  ≤184  >184 to 163 | | Cortisol AM  ≥618 | |
|  |  |  | **Normal TSH**  038 to 5.33 | TSH  >38 to 5.32 |  | **AM**  N:185 to 617 | |  |  |  |  |
|  |  |  | **T4 pmol/l** | T4  ≤ 7.2 | T4  ≥ 16.4 | **Cortisol (PM)**  ≤ 276 | | Cortisol PM  ≤ 276 | | Cortisol PM  ≥ 277 | |
|  |  |  | N:7.2 to 16.4 |  |  |  |  |  |  |  |  |
| **END OF INPATIENT DATA CAPTURING SECTIONS** | | | | | | | | | | | |
| **BEGINNING OF OUTPATIENT FOLLOW UP SECTION FIR PATIENTS WHO HAD CATATONIA** | | | | | | | | | | | |
| **K.**  **Follow-up**  **Period**  **ONLY** | **Date of Discharge** | | **1 month** | **2 months** | **3months** | | **Other** | | | | |
| Please tick the applicable box | Recurrence of Catatonia?  Yes  No | | Recurrence of Catatonia?  Yes  No | Recurrence of  Catatonia?  Yes  No | Other? | | | | | | |
|  | Re-  Admission?  Yes  No | | Re-Admission?  Yes  No | ReAdmission?  Yes  No | Other? | | | | | | |
| **L.**  **Please describe (in your own words) your experience/ of the catatonic episode in terms of your thoughts, feelings and behaviour** | **L. Uyacelwa uchaze (ngawakho amazwi) ngamava akho ngexesha ubune catatonia ngokwengcinga zakho, indlela obuziva ngayo nezinto obuzenza.** | | **PARTICIPANT RESPONSE RECORDED VERBATUM (USE AUDIO RECORDER)**  **Thoughts**  **Feelings**  **Behaviour** | | | | | | | | |

# APPENDIX B: INFORMED CONSENT FORM INFORMED CONSENT

**INKCAZELO NGOPHANDO LWE-CATATONIA**

Le yindlela esikwazisa ngayo ngophando esilwenzayo kweli candelo ulaliswe kulo, olumalunga ne-catatonia. Kolu phando sijonge ukubala ukuba bangaphi abantu abafunyanwa yi-catatonia kubantu abalaliswe apha. Ukuba ufuna ukwazi banzi ngolu phando, likhona iphepha ongalifumana elinenkcazelo ebanzi. nongalicela kumongikazi okanye ugqirha. Awunyanzelekanga uthathe inxaxheba ukuba awufuni. Uzakuqhubekeka ulunikwe unyango lwako lwesiqhelo nokuba unokukhetha ukungavumi ukungenela olu phando.

I-catatonia sisigulo esiye sibangele ukuphazamiseka kwindlela umntu ashukuma ngayo apha emzimbeni. Kwabanye abantu sibangela ukuba umzimba lo ucothe kakhulu okanye ungakwazi kushuma, umntu azive eqinile, athi nokuba uyafuna ukushumisa umzimba wakhe njengesiqhelo angakwazi. Ngelinye ixesha i-catatonia iyakwazi ukubangela ukuba umntu athi ngoku sele eqalile ukushukuma esithi wenza into ethile, suka umzimba lo uqine, aphethe amalungu omzimba afana neengalo, izandla, imilenze okanye iinyawo zilenga emoyeni angakwazi ukuyigqibezela laa nthsukumo ebeyiqalile. I-catatonia iyakwazi ukuphinda ibangele intshukumo engaphaya kunesiqhelo, aphethe umntu eshuku-shukuma kakhulu, angahlali ndawonye okanye angazinzi. Abanye baye bazule ndawoninye, abanye baqhwabe izandla unomphelo okanye banqwale kungenjalo baninike intloko into engapheliyo.

| **1.Yintoni injongo yolu phando ndigqiba kukucacisela ngalo?** | **1^st^ Attempt** | **2^nd^ Attempt** | **3^rd^ Attempt** |
| --- | --- | --- | --- |
|  | Andazi  0 | Andazi  0 | Andazi  0 |
|  | Lumalunga ne catatonia  1 | Lumalunga necatatonia  1 | Lumalunga necatatonia  1 |
|  | Lumalunga nokubala bangaphi abantu abafunyanwa yicatatonia  2 | Lumalunga nokubala bangaphi abantu abafunyanwa yicatatonia  2 | Lumalunga nokubala bangaphi abantu abafunyanwa yicatatonia  2 |
| **2.Ndingazikhethela ukuba ndiyafuna ukuthatha inxaxheba kolu phando?** | Andazi  0 | Andazi  0 | Andazi  0 |
|  | Ndinako ukuthi hayi okanye ndithi ewe  1 | Ndinako ukuthi hayi okanye ndithi ewe  1 | Ndinako ukuthi hayi okanye ndithi ewe  1 |
|  | Ndinelungelo lokuzikhethela ukuba ndiyavuma okanye andivumi ukuthetha inxaxheba kolu phando  2 | Ndinelungelo lokuzikhethela ukuba ndiyavuma okanye andivumi ukuthetha inxaxheba kolu phando  2 | Ndinelungelo lokuzikhethela ukuba ndiyavuma okanye andivumi ukuthetha inxaxheba kolu phando  2 |
| **3. Ndisayikhumbula yonke lenkcazelo ndiyifumeneyo ngolu phando.** | Hayi andisayikhumbuli  0 | Hayi Andisayikhumbuli  0 | Hayi Andisayikhumbuli  0 |
|  | Ewe Ndiyayikhumbula kancinci  1 | Ewe Ndiyayikhumbula kancinci  1 | Ewe Ndiyayikhumbula kancinci  1 |
|  | Ewe ndiyikhmbula yonke  2 | Ewe ndiyikhmbula yonke  2 | Ewe ndiyikhmbula yonke  2 |
| **4. Ukuba andivumi loo nto ingaluphazamisa unyango endilunikwayo?** | Ewe  0 | Ewe  0 | Ewe  0 |
|  | Mhlawumbi  1 | Mhlawumbi  1 | Mhlawumbi  1 |
|  | Hayi ayinakuluphazamisa unyango endilufumanyo  2 | Hayi ayinakuluphazamisa unyango endilufumanyo  2 | Hayi ayinakuluphazamisa unyango endilufumanyo  2 |
| **TOTAL ( 0 - 8)** |  |  |  |

**A SHORT SUMMARY ABOUT THE CATATONIA STUDY**

This is a short summary to tell you about the research into catatonia that we are undertaking in the mental health unit you have been admitted in. In this study we want to find out how many of the people admitted here are affected by catatonia. If you would like more detailed information about the study, you can ask for the more detailed information leaflet from your nurse or doctor. You have the right to choose whether you would like to be part of this study or not. You wil get your treatment as usual whether you agree to take part in the study or not.

Catatonia is a condition that affects the way a person moves his whole body or body parts. In some people it slows down the body considerably to the point where some will stop moving completely, causing the person to feel very stiff such that they are unable to move even when they want to. This may lead to a person remaining in one position for a very long time (whether sitting or standing) to the point of many minutes or even hours. Catatonia can also cause a person to appear frozen even after initiating a particular action, resulting in body parts like legs, arms hands or feet being frozen in awkward or unusual looking positions. Catatonia can also cause an abnormality of excessive movement which is more than normal. A person may show excessive movement that lasts up to many minutes or hours with a seeming inability to stay still.

| **1.What is the study that I just explained to you about?** | **1^st^ Attempt** | **2^nd^ Attempt** | **3^rd^ Attempt** |
| --- | --- | --- | --- |
|  | I don’t know  0 | I don’t know  0 | I don’t know  0 |
|  | It is about catatonia  1 | It is about catatonia  1 | It is about catatonia  1 |
|  | It ia about how many people get affected by catatonia  2 | It ia about how many people get affected by catatonia  2 | It ia about how many people get affected by catatonia  2 |
| **2.I can choose whether I want to take part or not in this study** | **** | **** | **** |
|  | I can say yes or no  1 | I can say yes or no  1 | I can say yes or no  1 |
|  | I have a right to choose whether to take part in the study or not  2 | I have a right to choose whether to take part in the study or not  2 | I have a right to choose whether to take part in the study or not  2 |
| **3. I can remember all the information you gave me about the study** | No I cannot remember it  0 | No I cannot remember it  0 | No I cannot remember it  0 |
|  | Yes I remember a little bit  2 | Yes I remember a little bit  2 | Yes I remember a little bit  2 |
|  | Yes I remember all/most of it  2 | Yes I remember all/most of it  2 | Yes I remember all/most of it  2 |
| **4. If I refuse to take part in the study, will that interfere with the treatment I am going to receive?** | Yes  0 | Yes  0 | Yes  0 |
|  | Maybe  1 | Maybe  1 | Maybe  1 |
|  | No, It cannot interfere  2 | No, It cannot interfere  2 | No, It cannot interfere  2 |
| **TOTAL ( 0 - 8)** |  |  |  |

**APPENDIX C: INFORMED CONSENT FORM FOR A STUDY ON CATATONIA**

Dear Participant or Relative

We are requesting your consent to enroll you in a study on catatonia and how the symptoms respond to the treatment you are going to be given.

**YES, I AGREE TO BE ENROLLED**

I ………………………………………………………………………………………………......

agree in **voluntarily** taking part in the study as explained to me by the doctor/ nurse

# OR

(In cases where the patient is incapable of giving consent but is not opposed to taking part in the study, then a relative or custodian may provide informed consent by also signing below)

I …………………………………… being the of

……………………………………… **willingly** agree that he/she may take part in the study which has been explained to us by the doctor/ nurse

Signature of participant/relative/ custodian:

…………………………………………………………………………………..

Signed by …………………………………… at on the

………… of 2019

**NO, I DO NOT AGREE TO BE ENROLLED**

I

………………………………………………………………………………………………......

do not agree in taking part in the study as explained to me by the doctor/ nurse OR

I …………………………………… being the of

……………………………………… do not agree that he/she may take part in the study which has been explained to us by the doctor/ nurse

## Signature of participant/relative/ custodian:

***……………………………………………………………………***

***FOR OFFICE USE ONLY: ASSESSMENT OF CAPACITY TO CONSENT BASED ON UBACC***

| ***Does the patient….***   1. ***Understand the information relevant to the decision?*** 2. ***Retain the information long enough to consider it?*** 3. ***Weigh the information as part of the decision-***   ***making process?***   1. ***Communicate their decision in some way?*** | ***Yes***  ***…………***  ***………….***  ***…………..***  ***…………..*** | ***No***  ***………..***  ***………..***  ***………..***  ***………..*** |
| --- | --- | --- |

*Please Note: Should there be a no answer to any of the 4 questions above then the patient lacks capacity to consent and a relative or custodian may then be requested to provide informed consent.\*

Isaynwe e…………………………… ngomhla we………….. kwinyanga ye ku

2019

Utyikitya apha wena okanye umzali okanye isizalwane

………………………………………………………………………

*Please Note: Should there be a no answer to any of the 4 questions above then the patient lacks capacity to consent and a relative or custodian may then be requested to provide informed consent .*

# APPENDIX D. MORE IN-DEPTH STUDY INFORMATION LEAFLET/ INCWADANA ECHAZA NGOLU PHANDO

# INFORMATION LEAFLET (XHOSA)

**Iphepha lokwazisa umthathi-nxaxheba kuphando lwesigulo i-Catatonia**

Mthathi-nxaxheba obekekileyo *okanye* Mzali okanye sizalwane esibekekileyo

Ngale ncwadi sikwazisa malunga ngophando nzulu oluqhutywa ziinzululwazi ezifuna ukufunda nzulu ngesigulo ekuthiwa yi-catatonia kweli ziko lempilo. Unyango obuhleli uzakulufumna alusayi kutshintsha okanye luphazamiseke wakuthatha inxaxheba kolu phando.

**Yintoni i-*catatonia*?**

I-catatonia le sisigulo esiye sibangele ukuphazamiseka kwindlela umntu ashukuma ngayo apha emzimbeni. Kwabanye abantu sibangela ukuba umzimba lo ucothe kakhulu okanye ungakwazi kushuma, umntu azive eqinile, athi nokuba uyafuna ukushumisa umzimba wakhe njengesiqhelo angakwazi. Ide ibangele loo nto ngelinye ixesha ukuba umntu aphethe ehleli ndawoninye okanye emile ndawoninye de kugqithe imizuzu emininzi okanye iiyure zibe liqela. Iyakwazi nokubangela ukuba umntu angakwazi ukuphuma kwasebhedini, angakwazi kuzityisa, angakwazi kuzihlamba, asoloko elele ebhedini okanye ehleli esitulweni.

Kwelinye icala i-catatonia iyakwazi ukubangela ukuba umntu athi ngoku sele eqalile ukushukuma esithi wenza into ethile, suka umzimba lo uqine, aphethe amalungu omzimba afana neengalo, izandla, imilenze okanye iinyawo zilenga emoyeni angakwazi ukuyigqibezela laa nthsukumo ebeyiqalile. Intamo nentloko nazo ziyakwazi ukuphetha zikekele ngenxa yoku kuqina komzimba kuvela ngesiquphe.

Okokugqibela, i-catatonia iyakwazi ukuphinda ibangele intshukumo engaphaya kunesiqhelo, aphethe umntu eshuku-shukuma kakhulu, angahlali ndawonye okanye angazinzi. Abanye baye bazule ndawoninye, abanye baqhwabe izandla unomphelo okanye banqwale kungenjalo baninike intloko into engapheliyo. Iyakwazi nokuvela ngokuba omnye umntu abetha-bethe amanqindi emoyeni, omnye athi nokuba usebhedini kube ngathi unyomfa ibhaysikili into engapheliyo. Babakhona nabaphetha bethetha into enye, okanye benze isikhalo esiphindaphindwayo okanye nayiphina intsholo abaye bayiqhube imizuzu emininzi okanye iiyure zide zibe liqela. Bakhona ke nabanye abaye balinganise loo nto ithethwa ngumntu ophambi kwabo kungenjalo balinganisa loo nto bayibona isenziwa ngumntu ophambi kwabo.

**Ibangelwa yintoni i-*catatonia*?**

I-catatonia iyakwazi ukubangelwa zizigulo ezithile zengqondo kungenjalo nezinye izigulo zomzimba ziyakwazi ukuhamba ne-catatonia. Ingxaki esiye siyifumane thina boogqirha neenzululwazi kukungazi xa siqala ukumbona umntu onale catatonia ukuba ingaba eyakhe ibangelwa sisigulo sengqondo na okanye sesomzimba kusini na. Yiloo tno side sabona ukuba kungakuhle ukuba sinokuyiphonononga nzulu le ngxaki.

# Luqulethe ntoni olu phando?

Sijonge ekubeni wonke umntu oze kulaliswa kweli candelo, ahlolwe, kukhangelwe ukuba akahlaselwanga ziimpawu ezithile zale-catatonia kusini na. Wothi uhlolwe ngugqirha wakho ebehleli ezakuhlola kakade. Ukuba zikhona iimpawu ezithile ugqirha acinga ukuba uziqaphele apha kuwe, usenakho ukubuza ngazo umzekelo mhlawumbi ukuba ziqale nini, njani, kwaye ingaba uyaqala ukuba nazo na njalonjalo. Uzakube phofu ebhala loo nto umxelela yona. Apha ekubhaleni kwakhe kodwa akazokulibhala igama lakho nokuba ungubani kwaye uhlala phi. Oku kuhlolwa nokubuzwa nge-catatonia kungathatha imizuzu emihlanu ukuya kweli shumi kuphela. Ulwazi esiluqokelelayo ngawe kukuba nje iimpawu zesi sigulo unazo na kwaye nale mibuzo sesiyikhakanyile kuphela.

Zimbini izinto esifuna ukuziqwalasela kolu phando nge-catatonia:

1. Ingaba bangaphi abantu abafunyanwa sesi sigulo kule ngingqi?
2. Ingaba zikhona izinto ezingunobangela wokuba abanye abantu bafunyanwe sesi sigulo abanye basinde, mhlawumbi njengobubudala bomntu, isini okanye ezinye izigulo zomzimba?

Nayiphi na into esinokuyifunda eyongezelela kulwazi esele sinalo ngesi-sigulo ingasinceda kakhulu ekubeni sikwazi ukusinyanga ngcono kwixa elizayo. Ngako oko ubukho bakho nokuthatha kwakho inxaxheba kolu phando kuya kunceda abantu abaninzi abanokuthi bafunyanwe sesi sigulo.

Alukho olunye uvavanyo oza kulwenza oludibene nolu phando. Naluphi na olunye uvavanyo okanye unyango ozakuthi ulufumane emva kokuba umongikazi okanye ugqirha egqibile ukukuhlola, lunyango lwesiqhelo obuhleli uzakulufumana kakade kugirha wakho.

# Ukuba ndifunyaniswe ndinazo imipawu ze-*catatonia* loo nto ithetha ukuthini?

Ukuba ufunyaniswe unazo ezinye zezi mpawu ze-catatonia, ugqira wakho wokunika unyango lakho lwesiqhelo okanye enze uvavanyo ebehleli ezakulwenza kakade olunxulumene nempilo yakho.

# Kuza kwenziwa ntoni ngeziphumo zolu phando?

Iziphumo zolu phando zizakudityaniswa zibhalwe kufndiswe abanye oogqirha neenzululwazi malunga nesi sigulo, kwiinkomfa zoogqirha neenzululwazi.

# Ndithini ukuba ndinemibuzo?

Ukuba unemibuzo ungacela ukuthetha nogqirha wakho okanye umongikazi ozakube encedisa kolu phando.

**Siyabulela!** Sibulela kakhulu ngexesha lakho nokuzixhesha kwakho ngolu phando.

**INFORMATION LEAFLET ABOUT A STUDY OF CATATONIA**

Dear Participant / Parent/ Relative

This leaflet is provided to inform you about a study being conducted by researchers who would like to investigate a condition called catatonia at his health facility. The usual care you were going to get will not be changed or disturbed through taking part in this study.

# What is catatonia?

Catatonia is a condition that affects the way a person moves his whole body or body parts. In some people it slows down the body considerably to the point where some will stop moving completely, causing the person to feel very stiff such that they are unable to move even when they want to. This may lead to a person remaining in one position for a very long time (whether sitting or standing) to the point of many minutes or even hours. It can even cause some people to be bedridden, unable to feed themselves, or wash or attend to other daily needs.

Catatonia can also cause a person to appear frozen even after initiating a particular action, resulting in body parts like legs, arms hands or feet being frozen in awkward or unusual looking positions. The head and or neck may also be tilted at awkward angles. The change in movement can often occur suddenly.

Lastly catatonia can also cause an abnormality of excessive movement which is more than normal. A person may show excessive movement that lasts up to many minutes or hours with a seeming inability to stay still. Some people may pace up and down, others may clap or wave for long periods lasting minutes to hours, while others may show head nodding, head shaking, grimacing, etc. Some people have been seen to do shadow boxing or cycling movements even when lying down. It may also appear as repetitive speech of the same phrase, a cry or shout or other odd sound that can last for hours. Others may repeat what they hear around them non- stop or they may mimic actions of those around them as well.

# What causes catatonia?

Catatonia may be seen with a number of mental illnesses but it can also be associated with some other medical conditions. The problem we run into as doctors is when a person presents with the first time with this syndrome it may be difficult in the beginning to know what the underlying cause is i.e. whether the cause is a mental condition or another medical condition. This is why conducting research on catatonia is so important.

# What does this research involve?

We are looking at ensuring that everybody who is admitted into this unit s examined and screened for symptoms and signs of catatonia. Your admitting doctor will examine you as usual, which will include an initial screen for catatonia through examination only. Following this, a trained research assistant who is a nurse will proceed to do a full screen using a rating scale, to ensure that no other signs of catatonia were missed. If the research assistant finds any additional signs of catatonia, they will tell your treating doctor. In addition, the nurse may ask you questions like when did the symptoms start and how fast did they appear etc. She or he will note down you answers but will not include details like your name or your address which can identify who you are. This further screening by the nurse not expected to take longer than 5 to 10 minutes. The information to be collected for the study about your condition is about the signs and symptoms and the few questions already mentioned to do with the illness, nothing more. There are two questions we would like to investigate about catatonia:

1. How many people experience this condition in this area?
2. Are there particular characteristics that make some people more prone to it and others less vulnerable to it like age, gender or other medical conditions?

Whatever we can learn about this condition, over and above what we know already will help us to come up with improved ways to diagnose it and to treat it in future. Taking part in this research will therefore help many people in future who may also get this illness. There are no other tests you will be expected to take part in for this study. Whatever other tests or treatment interventions that follow will be those that your doctor would have undertaken anyway to help you manage your condition and get you better.

**If I am found to show some of the symptoms or signs of catatonia what does that mean?**

If you are found to have some signs and symptoms of catatonia, the research nurse will inform your treating doctor, so that your doctor can give you the appropriate and usual treatment for your condition. Your doctor may also decide to do more tests which would be what they would have done anyway even if you were not part of the study, in order to manage your condition.

# What will be done with the results of the study?

The results of the study will be collected and put together to present to scientific congresses so that other doctors and scientists can learn from them.

# What should I do if I have more questions?

If you have more questions, ask your treating doctor or the researcher, research assistant or nurse.

# Thank you!

Thank you very much for your patience and for spending the few minutes on this study.


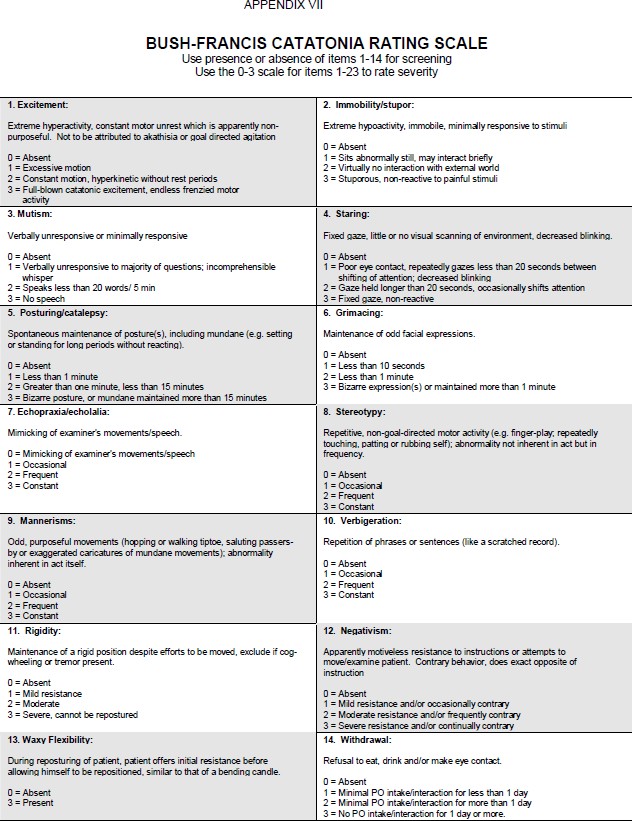


**APPENDIX E: THE BUSH FRANCIS SCALE**


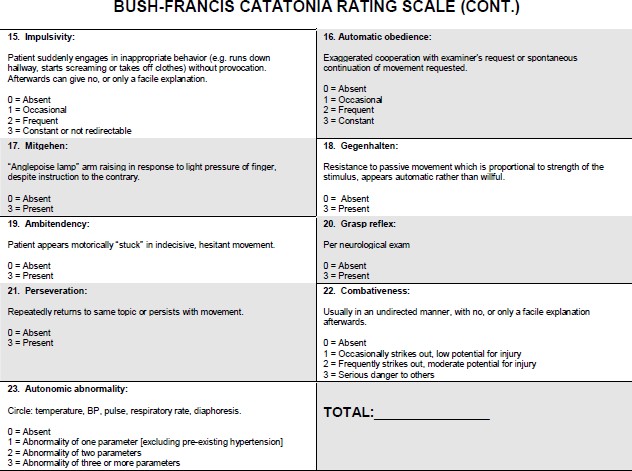


| **Is the Patient Catatonic ?** | **No because they have a score of less than 2)** | **Yes because they have a score of 2 and above** |
| --- | --- | --- |

**Standardized examination for catatonia.** The method described here is used to complete the 23- item Bush-Francis Catatonia Rating Scale (CRS) and the 14-item Catatonia Screening Instrument (CSI). Item definitions on the two scales are the same. The CRS measures the severity of 23 signs on a 0- 3 scale, while the CSI measures only the presence or absence of the first 14 signs.

Ratings are to be made solely on the basis of observed behaviour during the examination with the exception of completion of the items for 'withdrawal' and autonomic abnormality', which may be based on directly observed behaviour and for chart documentation. As a general rule, only rate items which are clearly present. If uncertain as to the presence of an item, rate the item as '0'.

|  | **Procedure** | **Examines** |
| --- | --- | --- |
| 1 | Observe patient while trying to engage in a conversation | Activity level, Abn movements Abn speech |
| 2 | Examiner scratches head in exaggerated manner | Echopraxia |
| 3 | Examine arm for cogwheeling. Attempt to reposture, instructing patient to "keep your arm loose" - move arm with alternating lighter & heavier force. | Negativism, Waxy flexibility |
| 4 | Ask patient to extend arm. Place one finger beneath hand and try to raise slowly after stating, "Do NOT let me raise your arm". | Passive obedience |
| 5 | Extend hand stating "Do NOT shake my hand". Gets stuck trying to do both. | Motorically stuck |
| 6 | Reach into pocket and state, “Stick out your tongue, I want to stick a pin in it". | Automatic obedience |
| 7 | Check for grasp reflex. | Grasp reflex |
| 8 | Check chart for reports of previous 24-hour period. In particular check for oral intake, vital signs, and any incidents. |  |
| 9 | Observe patient indirectly, at least for a brief period, each day. |  |
|  |  | |

References:

Bush G, Fink M, Petrides G, Dowling F, Francis A. Catatonia. I. Rating scale and standardized examination.

*Acta Psychiatr Scand*. 1996;93(2):129–136.

**APPENDIX F: BASED ON DSM-5 DIAGNOSTIC CRITERIA FOR CATATONIA**

| **Symptom (DSM-5)** | **Corresponding item on BFCRS** | **Absent i.e. None (0)** | **Present (1)** |
| --- | --- | --- | --- |
| 1. Stupor   (No Psychomotor activity, not actively reacting to environment) | Item 2 |  |  |
| 1. Catalepsy   (Passive induction of a posture held against gravity) | Item 5 |  |  |
| 1. Waxy flexibility   (slight and even resistance to positioning by examiner) | Item 13 |  |  |
| 1. Mutism (no or very little verbal response) | Item 3 |  |  |
| 1. Negativism (opposition or no response to instructions or external stimuli) | Item 12 |  |  |
| 1. Posturing (spontaneous and active maintenance of a posture against gravity) | Item 5 |  |  |
| 1. Mannerism (odd and out of context carrying out of normal, usually goal directed actions) | Item 9 |  |  |
| 1. Stereotypy (repetitive, abnormally frequent, non-goal directed movements) | Item 8 |  |  |
| 1. Agitation (not influenced by external stimuli) | Item 1 |  |  |
| 1. Grimacing | Item 6 |  |  |
| 1. Echolalia(mimicking another’s speech) | Item 7 |  |  |
| 1. Echopraxia (mimicking another’s actions) | Item 7 |  |  |
| **TOTAL (0 to 12)** |  |  |  |
| **Is the Patient Catatonic ?** |  | **No because they have a score of less than 3)** | **Yes because they have a score of 3 and above** |

*Reprinted with permission from the Diagnostic and Statistical Manual of Mental Disorders, Fifth Edition, (Copyright 2013). American Psychiatric Association.*
